# The Effects of Abdominal Draw-In Maneuvers Combined with Blood Flow Restriction on the Transverse Abdominis in University Students with Sedentary Lifestyles

**DOI:** 10.3390/life15060965

**Published:** 2025-06-17

**Authors:** Yueh-Ling Hsieh, Tzu-Yu Weng, Nian-Pu Yang, Yu-Liang Lai, Andy Chien

**Affiliations:** 1 Department of Physical Therapy, Graduate Institute of Rehabilitation Science, China Medical University, Taichung 404, Taiwan; sherrie@mail.cmu.edu.tw (Y.-L.H.); chuyu0608@gmail.com (T.-Y.W.); 2 National Defense Medical Center, School of Medicine, Taipei 114, Taiwan; ruby901201@gmail.com; 3 Department of Physical Medicine and Rehabilitation, China Medical University Hsinchu Hospital, Hsinchu 302, Taiwan; 4 Department of Physical Medicine and Rehabilitation, China Medical University Hospital, Taichung 404, Taiwan; 5 Department of Biomedical Engineering, College of Medicine and College of Engineering, National Taiwan University, Taipei 106319, Taiwan

**Keywords:** core stabilization exercise, transversus abdominis, blood flow restriction training (BFRT), physical inactivity

## Abstract

The abdominal drawing-in maneuver (ADIM) is one of the most valuable exercises for explicitly targeting and strengthening the transversus abdominis (TrA), a key muscle in the deep core. However, using the ADIM for the selective training of the transverse abdominis can be challenging for certain individuals. This study investigated the effects of combining ADIM with blood flow restriction (BFR) training on TrA strengthening in sedentary university students. Forty university students with sedentary lifestyles (mean age: 23.28 ± 2.468 years; range 20–25 years) were randomly assigned to an ADIM+BFR group and a control group (ADIM only). Both groups underwent 25 min ADIM training sessions twice weekly for four weeks. Ultrasound measurements assessed TrA thickness, contraction ratio, and preferential activation. Core stability, strength, and endurance were evaluated using the double leg lowering, abdominal strength, and abdominal static endurance tests. The ADIM+BFR group showed significant improvements in TrA thickness, contraction ratio, and preferential activation compared to the control group following the four-week training intervention (*p* < 0.05). The ADIM+BFR group demonstrated improved core stability and enhanced abdominal strength and endurance compared to the control group (*p* < 0.05). The results support the effectiveness of ADIM+BFR training in enhancing TrA contraction and activation in sedentary university students. This approach also improves core stability, strength, and endurance. BFR provides a novel and readily applicable method for promoting TrA activation during ADIM training.

## 1. Introduction

The global surge in sedentary lifestyles, particularly among university students, is a pressing health concern due to their increased screen times and sitting durations comparable to or exceeding those of office workers [1,2,3,4]. With increasing screen time and prolonged static sitting, this population is at a heightened risk of developing weakened deep trunk muscles, leading to fatigue, dysfunction, poor posture, reduced lumbar stabilization and low back pain [5,6]. While research has highlighted the adverse effects of sedentary behavior [7], few studies have explicitly focused on the impact of strengthening deep trunk muscles in this population. This is particularly alarming given it is well established that bad habits formed in young adulthood often persist into later life without early and effective intervention.

The transversus abdominis (TrA), in concert with the lumbar multifidus, constitutes a core musculature that exerts a compressive force on the spine, providing essential lumbar stability [8,9]. Research indicates that increasing the thickness and recruitment of these muscles through the performance of the abdominal drawing-in maneuver (ADIM), which selectively activates the TrA, correlates with enhanced spinal support, essential for maintaining spinal stability and preventing low back pain [10,11]. However, compiling evidence has shown that the TrA is not activated autonomously in the sedentary population due to poor neuromuscular control and the reduced baseline strength of the core muscles arising from muscle inhibition and lack of use. Despite the well-known benefits of core muscle activation training in the prevention and treatment of low back pain, it is also recognized that the best strategies to teach individuals to effectively perform the ADIM remain debatable [12]. Accurately performing the ADIM is challenging even in healthy individuals, primarily due to the difficulty in perceiving, palpating, and localizing the TrA, the deepest layer of all abdominal muscles [13,14,15]. Needless to say, sedentary individuals may find effectively performing the ADIM even more challenging due to their weakened core musculature and impaired postural stability.

Accumulating evidence indicates that blood flow restriction (BFR), when combined with low-load resistance exercise, elicits significant hypertrophic and strength adaptations in skeletal muscle. Investigations have further demonstrated its potential to mitigate muscle atrophy during periods of disuse [16,17]. Although BFR has shown promise in augmenting strength and hypertrophy in extremity muscles, an optimal implementation strategy for BFR protocols in trunk core musculature remains elusive. Furthermore, venous thromboembolism (VTE) is a reported risk of BFR training, though the incidence is very low when proper protocols are followed, and its implications for ADIM remain largely unknown.

Given the significant health implications of sedentary lifestyles among university students associated with poor deep core muscle activation, which can contribute to postural and back pain issues, this study aimed to investigate the effects of combining BFR with the ADIM in strengthening deep trunk muscles, particularly the TrA, and improving core stability through the facilitation of better lumbar spine stability, improved posture, and a reduced risk of low back pain within this population. Based on preliminary evidence, it was hypothesized that combining the ADIM with BFR would lead to greater improvements in TrA activation and overall core stability than ADIM exercise alone.

## 2. Materials and Methods

### 2.1. Participants and Study Design

The participants were university undergraduate students aged 20–25 years (23.28 ± 2.46 years) with a normal BMI (18.5–24.99, mean 22.67 ± 3.32) from the College of Allied Health Sciences. A balanced gender ratio was maintained in the sample. Participants were excluded if they had a history of chronic low back pain or any acute low back pain, cardiovascular disease, abdominal surgery, spinal surgery, spinal disorders, pre-existing clotting issues, or pregnancy. Sedentary behavior, defined as activities with minimal energy expenditure above resting levels (1.0–1.5 METs) such as sleeping, sitting, reclining, television viewing, and other screen-based entertainment, was assessed in university students. Based on a previously published methodology [18], daily sitting duration was measured separately for weekdays and weekends. Daily averages were computed as weighted averages, with weekdays weighted 5/7 and weekends 2/7. Participants with a daily sitting duration exceeding 480 min were considered sedentary.

Participants were informed about the study’s requirements, potential risks, and benefits. They were also asked to be injury-free during testing. Written informed consent was obtained from all participants and their parents (if applicable) involved in the study. Written informed consent was obtained from the participants to publish this paper. The study was conducted in accordance with the Declaration of Helsinki, and approved by the Institutional Review Board of the China Medical University (reference no. CRREC-109-007; NCT04270695; date approved: 5 February 2020). In the absence of comparable data, a moderate effect size (ηp^2^ = 0.25, approximately equivalent to Cohen’s *d* ~0.5) was assumed as a conservative estimate of the expected group × time interaction effect based on the proposed implementation of a two-way repeated-measures ANOVA. A plausible effect size of 0.5 (medium level) was selected based on related research in the lower limb BFR literature, which often reports moderate improvements. Subsequently, the power analysis indicated that a total of 40 participants (20 per group) would yield a power >0.08 at an α of 0.05 to detect a significant difference between the two groups (G*Power, version 3.1.9.7). After completing all the pre-training assessments, participants were randomly assigned to either the experimental group, who performed ADIM exercises combined with BFR on the abdomen (ADIM+BFR, *n* = 20), or the control group, who performed ADIM exercises combined with sham-BFR (ADIM group, *n* = 20), with gender matched within each group. Both groups trained twice weekly for four weeks, with assessments at baseline (Week 0), post-training (Week 4), and follow-up (Week 5). Outcomes included sonographic measurements and double leg lowering, abdominal strength, and endurance tests.

### 2.2. Intervention

During the same session, a participant from the ABR+BFR group and a participant from the ADIM (control) group engaged in ADIM training concurrently.

#### 2.2.1. Abdominal Drawing-In Maneuver

All ADIM trainings were conducted under the guidance of the same therapist. Participants lay supine on a therapy table with bent knees, aligning their nose, sternum, and navel. The therapist instructed participants to inhale, exhale, and gently draw their navel toward their spine. During the movement, ribcage rotation should be downward, the chest should remain stable, pelvic movement should be minimized, and plantar foot force should be reduced. The therapist provided consistent verbal cues: “Inhale deeply, then exhale and draw your navel towards your spine; hold for 10–15 s” [19,20]. Participants were also instructed to use visual feedback from a stabilizer’s pressure gauge to maintain the appropriate pressure during ADIM execution. Participants performed 20 repetitions of the ADIM with a 1 min rest between each repetition. Each intervention lasted about 25 min twice weekly for four weeks, totaling eight sessions.

#### 2.2.2. Combined Abdominal Drawing-In Maneuver and Blood Flow Restriction Training

**Figure 1:**
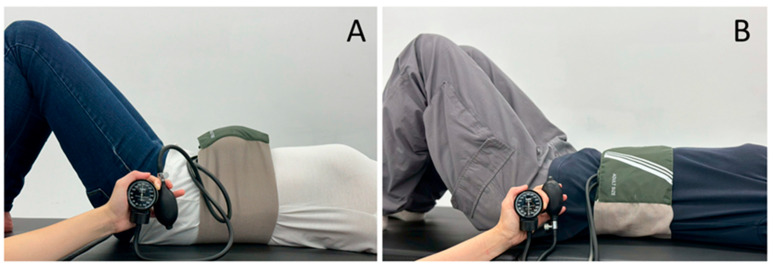
The experimental setup for the ADIM + BFR intervention showcased an inflatable nylon pressure cuff applied during knee flexion (**A**) and the ADIM + sham − BFR (control) intervention used a non-inflated cuff (**B**).

For the ADIM+BFR group, an inflatable nylon pressure cuff (100 cm × 20 cm, P-106NXXL+P-107ADT) was wrapped around the 12th rib and inflated to 160 mmHg using a mercury sphygmomanometer (CK-201) during ADIM. The ADIM (control) group had the same pressure cuff placement but was only inflated to 10 mmHg with the air valve open, creating a realistic sham comparison; i.e., this allowed participants to perceive the inflation of the pressure cuff without reaching the appropriate treatment pressure required. With the air valve remaining open, this gradually allowed the pressure gauge to return to 0 mmHg after a couple of ADIM. Both groups followed the same verbal cues and performed the ADIM with the same intensity and duration (Figure 1). It is acknowledged that no prior studies have implemented BFR in trunk applications; therefore, there were no existing BFR pressures to reference for this study. However, 160 mmHg was chosen as it is roughly 1.3 times a typical resting systolic blood pressure and falls within moderate occlusion ranges commonly used in BFR training of the lower limbs (100–180 mmHg). During preliminary trials, 160 mmHg was well-tolerated by participants whilst achieving the desired occlusive effect, as monitored by the Doppler, without causing pain or numbness. All participants were continuously monitored for any sudden discomfort to mitigate any potential risks.

### 2.3. Outcome Measurements

#### 2.3.1. Demographics

The following baseline data were collected: age, gender, height, weight, years of education, sedentary behavior (minutes per week), and duration of regular exercise (minutes per day) recorded at the baseline phase.

#### 2.3.2. Ultrasound Image Analysis

Abdominal muscle thickness was assessed using a Terason t3000 ultrasound system (Teratech, Burlington, MA, USA) equipped with a linear probe (brightness-mode, 12L5, Part #10-3276, Burlington, MA, USA). This probe has a frequency range of 5–12 MHz and a maximum penetration depth of 5 cm. The transducer was positioned horizontally with a slight rotation in the caudal direction midway between the lowest rib and the anterior iliac crest. The TrA, internal oblique (IO), and external oblique (EO) muscles and their movements during ADIM contraction had to be depicted in the ultrasound image. All ultrasound scans were performed by a rehabilitation physician, with 10 years of experience in musculoskeletal ultrasound imaging, who was blinded to the study group assignment. Ultrasound images of the abdominal muscles were acquired during muscle relaxation and at the 5 s mark of the three ADIM repetitions. The thickness of the TrA, IO, and EO muscles and the total abdominal muscle thickness were measured using the ultrasound imaging software. Measurements were taken 3 cm medial to the tip of the TrA at its caudal end and of the thoracolumbar fascia, from inferior to superior, to calculate the thickness of the TrA, IO, EO, and total muscle thickness, respectively. The in-built caliper and measurement software of the machine were used for the measurements. Three senior researchers independently measured the ultrasound images to obtain average values. The intraclass correlation coefficient (ICC) for the inter-rater reliability of this measurement technique was more significant than or equal to 0.95, as reported in the data analysis. From the above data, the following indices were calculated [21,22]: (1) contraction ratio (CR) = contracted thickness/resting thickness of the selective muscle; (2) contraction difference (Diff) = resting thickness − contracted thickness of the selective muscle; (3) preferential activation ratio (PAR) of a selective muscle = preferential activation ratio = (selective muscle contracted thickness/TrA + EO + IO contracted thickness) − (selective muscle resting thickness/TrA + EO + IO resting thickness).

#### 2.3.3. Abdominal Muscle Performance Assessments

Double Leg Lowering Test

The double leg lowering test (DLLT) assessed core abdominal stability [23]. Participants were positioned supine on a bed with their hips flexed at 90 degrees and knees extended. A Stabilizer Pressure Biofeedback device (Chattanooga Group Inc., Hixson, TN, USA) was placed at the lower back and inflated to 40 mmHg to maintain pelvic posterior tilt. Following a practice trial, participants actively lowered their legs to the table while maintaining pelvic posterior tilt and the 40 mmHg pressure within the stabilizer. The angle between the legs and the table was recorded when the pressure dropped below 40 mmHg, indicating an inability to maintain pelvic stability. This test was repeated twice with a 5 min interval, and the average angle was recorded.

Abdominal Muscle Strength Test

The abdominal muscle strength test (AMST) was performed in a static sit-up position. Participants sat on a bed with their hands crossed in front of their chest, hips flexed at 70 degrees, knees slightly bent, and legs secured. A handheld dynamometer (microFET^®^3, Hoggan Scientific LLC, Salt Lake City, UT, USA) was placed on the participant’s sternum. Participants performed an isometric trunk flexion contraction for 5 s, and the peak force was recorded. The test was repeated twice, and the average was calculated.

Abdominal Endurance Test

Abdominal endurance was assessed using a static abdominal endurance test (AET). Participants leaned against a 60-degree inclined board with their hips and knees flexed at 90 degrees and hands crossed in front of their chest. The tester held the participant’s feet to prevent movement [24]. The test began when the board was 10 cm from the participant’s back. Participants maintained this position until their back was rounded and the angle between their back and the board was less than 60 degrees. The time in seconds was recorded as the measure of abdominal endurance.

Statistical Analysis

All measured data were expressed as means ± standard deviation (means ± SD). The Shapiro–Wilk test was used to assess the normality of the data distribution. For the within-subject factor (time), Mauchly’s Test of Sphericity was performed to examine the assumption of sphericity. If sphericity was violated, Greenhouse–Geisser corrections were applied. For normally distributed data, a 2 (group: ADIM+BFR and ADIM) × 2 (gender: male, female) × time (baseline, post-intervention, follow-up) mixed-design analysis of variance (ANOVA) was used to analyze the serial changes in the thickness of the EO, IO, and TrA on the ultrasound image outcomes. Group and gender were treated as between-subject factors, while time was treated as a within-subject (repeated measures) factor. Bonferroni post hoc tests were conducted to identify significant differences from baseline values. Independent *t*-tests determined differences between the ADIM+BFR and ADIM groups in both the female and male participants. All the statistical analyses were performed using SPSS (version 22.0, SPSS, Inc., Chicago, IL, USA), with statistical significance set at *p* < 0.05.

## 3. Results

### 3.1. Demographic Characteristics

**Figure 2:**
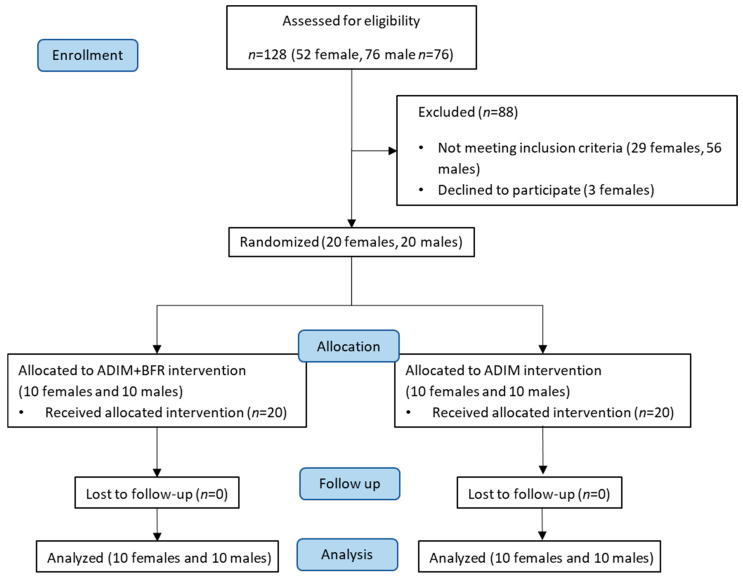
Study design and participant recruitment and allocation.

Participants were recruited, and the study was conducted between February 2023 and June 2024. The participants’ characteristics and sedentary time are described in Table 1. A total of 40 participants (20 per group) participated in the study (Figure 2). The range of the daily sitting duration was from 504 to 1538 min among these participants. No significant differences in demographic data, sedentary behavior (minutes/day), or duration of regular exercise (minutes/week) were evident between the two groups in each gender (Student’s *t*-test, all *p* > 0.05, Table 1).

### 3.2. Alternations in Muscle Thickness at Baseline, Post-Training, and at Follow-Up

**Figure 3:**
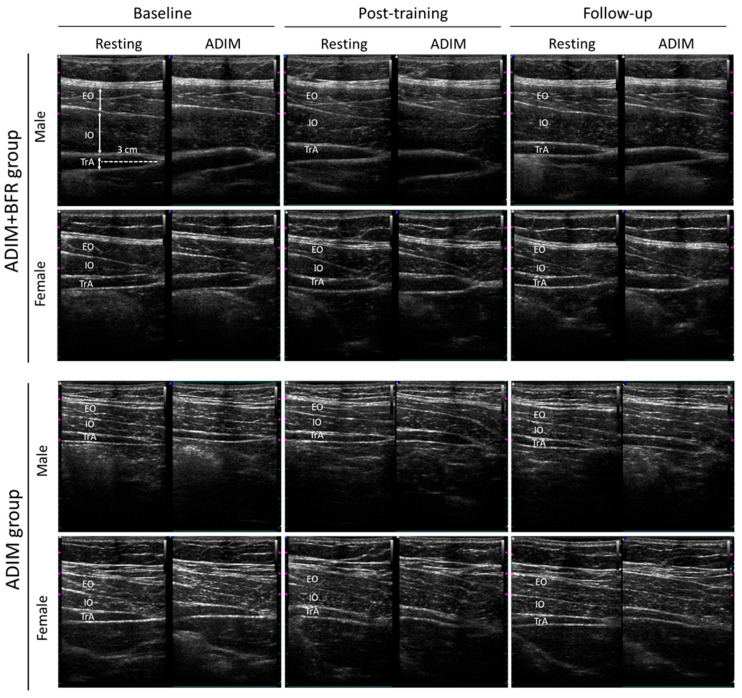
Ultrasound images of muscle thickness change across different time points and interventions. Changes in external oblique (EO), internal oblique (IO), and transversus abdominis (TrA) muscle thickness are shown through ultrasound imaging at baseline, post-training, and at follow-up during both resting and ADIM states. Specifically, the ADIM + BFR intervention significantly increased TrA thickness in both male and female participants.

**Figure 4:**
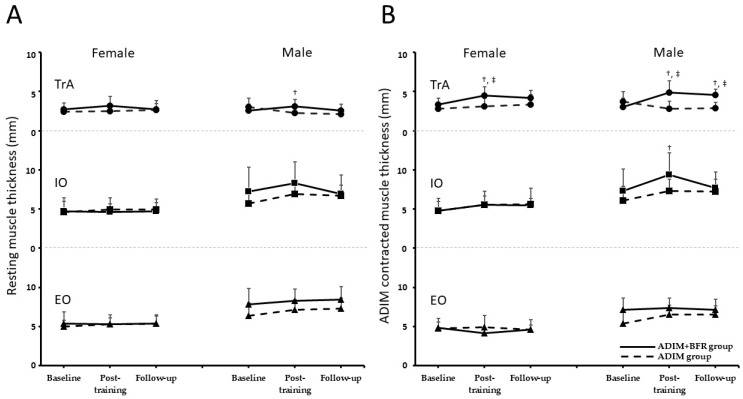
The changes in muscle thickness of the transversus abdominis (TrA), internal oblique (IO), and external oblique (EO) muscles across three time points—baseline, post-training, and follow-up—during both resting (**A**) and ADIM (**B**) states in ADIM+BFR and ADIM groups for both female and male participants. Statistically significant increases in TrA thickness were observed in the ADIM+BFR group post-training and at follow-up when compared to baseline (^‡^, *p* < 0.05) and the ADIM group (^†^, *p* < 0.05).

Figure 3 and Figure 4 illustrate muscle thickness changes at rest and during ADIM contraction, measured via ultrasound imaging at baseline, post-training, and at follow-up. A mixed-model two-way ANOVA was conducted to evaluate the effects of group and gender across the three time points for EO, IO and TrA muscle thickness. At rest, significant main effects of time (EO: F = 4.02, *p* < 0.05, ηp^2^ = 0.10; IO: F = 3.45, ηp^2^ = 0.09, *p* < 0.05) and gender (EO: *F* = 35.19, *p* < 0.05, ηp^2^ = 0.49; IO: F = 17.89, ηp^2^ = 0.33, *p* < 0.05) were observed for both EO and IO thickness. No significant interactions among time, gender, and group were found (*p* > 0.05). For TrA resting thickness, no significant main effects of time, group, or gender were observed (*p* > 0.05). However, a significant time-by-group interaction (F = 5.28, *p* < 0.05, ηp^2^ = 0.13) and a significant three-way interaction among time, gender, and group (F = 3.39, *p* < 0.05, ηp^2^ = 0.09) were found (Table 2). During ADIM contraction, a significant main effect of gender (F = 43.87, *p* < 0.05, ηp^2^ = 0.55) was observed for EO thickness. For IO thickness during ADIM, significant main effects of both time (F = 12.13, *p* < 0.05, ηp^2^ = 0.25) and gender (F = 19.04, *p* < 0.05, ηp^2^ = 0.04) were found. A significant group-by-gender interaction was observed for EO thickness (F = 4.59, *p* < 0.05, ηp^2^ = 0.11). For TrA thickness during ADIM, significant main effects of time (F = 6.96, *p* < 0.05, ηp^2^ = 0.16) and group (F = 14.17, *p* < 0.05, ηp^2^ = 0.28) were observed. Significant interactions were found between time and group (F = 14.34, *p* < 0.05, ηp^2^ = 0.29) and among time, gender, and group (F = 5.82, *p* < 0.05, ηp^2^ = 0.14) (Table 2).

Table 2 details the specific changes observed. The ADIM group exhibited no significant changes in resting or contracted muscle thickness for the EO, IO, or TrA across the three assessments (*p* > 0.05). Conversely, the ADIM+BFR group demonstrated significant time-dependent differences in contracted TrA thickness (females: F = 6.39, *p* < 0.05, ηp^2^ = 0.42; males: F = 12.20, *p* < 0.05, ηp^2^ = 0.58). Post hoc analysis revealed significant increases in contracted TrA thickness post-training compared to baseline for both female and male participants (*p* < 0.05, female: Cohen’s *d* = 1.10; male: Cohen’s *d* = 1.40). However, this increase was sustained only at follow-up for males (*p* < 0.05, male: Cohen’s *d* = 1.64). Significant differences in contracted TrA thickness between the ADIM+BFR and ADIM groups were observed post-training for both females (*t* = 2.70, *p* < 0.05, Cohen’s *d* = 1.21) and males (*t* = 3.71, *p* < 0.05, Cohen’s *d* = 1.66). Additionally, males in the ADIM+BFR group exhibited significantly greater resting TrA thickness post-training compared to the ADIM group (*t* = 2.67, *p* < 0.05, Cohen’s *d* = 1.19). Finally, a significant time-dependent increase in contracted IO thickness was observed exclusively in males (F = 7.16, *p* < 0.05, ηp^2^ = 0.44), specifically at the post-training assessment compared to baseline (*p* < 0.05).

### 3.3. Contraction Ratio, Contraction Difference, and Preferential Activation Ratio

Table 3 presents the muscle contraction abilities of the EO, IO, and TrA muscles during ADIM contractions, as indexed by the CR, Diff, and PAR across baseline, post-training, and follow-up assessments. A mixed-model two-way ANOVA was conducted to assess the effects of group and gender across the three time points. For the IO, significant within-time effects were observed for both CR (F = 4.46, *p* < 0.05, ηp^2^ = 0.11) and Diff (F = 4.91, *p* < 0.05, ηp^2^ = 0.12). For the EO, a significant within-time effect was observed for PAR (F = 11.36, *p* < 0.05, ηp^2^ = 0.24), along with a significant time-by-group interaction (F = 5.84, *p* < 0.05, ηp^2^ = 0.14). For the TrA, significant main effects of both time and group were observed for CR (time: F = 13.54, *p* < 0.05, ηp^2^ = 0.27; group: F = 12.77, *p* < 0.05, ηp^2^ = 0.26), Diff (time: F = 14.32, *p* < 0.05, ηp^2^ = 0.29; group: F = 17.22, *p* < 0.05, ηp^2^ = 0.32), and PAR (time: F = 10.32, *p* < 0.05, ηp^2^ = 0.22; group: F = 14.81, *p* < 0.05, ηp^2^ = 0.29). Significant time-by-group interactions were also found for TrA CR (F = 6.98, *p* < 0.05, ηp^2^ = 0.16), Diff (F = 8.02, *p* < 0.05, ηp^2^ = 0.18), and PAR (F = 5.39, *p* < 0.05, ηp^2^ = 0.13). A significant gender effect was observed for TrA PAR (F = 4.70, *p* < 0.05, ηp^2^ = 0.12) (Table 3).

The ADIM group showed no significant changes in CR, Diff, or PAR for any of the muscles (EO, IO, and TrA) across the three assessments (*p* > 0.05). In contrast, the ADIM+BFR group demonstrated time-dependent significant changes in CR, Diff, and PAR within the EO, IO, and TrA (*p* < 0.05) for both female and male participants. Specifically, females in the ADIM+BFR group exhibited significant improvements in the CR, Diff, and PAR of either the EO or IO at the post-training assessment compared to baseline (*p* < 0.05). Males in the ADIM+BFR group showed significant improvements in the CR, Diff, and PAR of the TrA at the post-training assessment compared to baseline (*p* < 0.05). These significant improvements in TrA contraction abilities were also observed at the follow-up assessment compared to baseline for both males and females in the ADIM+BFR group (*p* < 0.05). Finally, significant differences in TrA contraction abilities were observed between the ADIM+BFR and ADIM groups for both females and males at both the post-training and follow-up assessments (all *p* < 0.05).

### 3.4. Abdominal Muscle Performances

**Figure 5:**
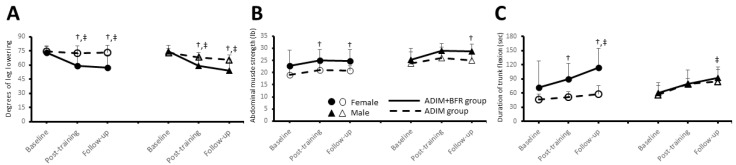
The changes in the double leg lowering test (DLLT, (**A**)), abdominal muscle strength test (AMST, (**B**)), and abdominal endurance test (AET, (**C**)) across baseline, post-training, and follow-up assessments in the ADIM+BFR and ADIM groups for both female and male participants. Statistically significant improvements in the DLLT were observed in the ADIM+BFR group compared to baseline post-training and at follow-up (^‡^, *p* < 0.05), with AET improvements significant only at follow-up (^‡^, *p* < 0.05). Independent *t*-tests demonstrated significant differences between the ADIM + BFR and ADIM groups in females for all three tests both post-training and at follow-up (^†^, *p* < 0.05), and in males for the DLLT post-training and at follow-up, and the AMST at follow-up (^†^, *p* < 0.05).

Figure 5 and Table 4 present the functional changes in abdominal muscles assessed at baseline, post-training, and at follow-up using the DLLT, AMST, and AET. A mixed-model two-way ANOVA was conducted to evaluate the effects of group and gender across the three time points for each test. For the DLLT, the analysis revealed significant main effects for both time (F = 36.05, *p* < 0.05, ηp^2^ = 0.5) and group (F = 15.52, *p* < 0.05, ηp^2^ = 0.30). A significant time-by-group interaction (F = 12.71, *p* < 0.05, ηp^2^ = 0.26) was also observed (Table 4). For the AMST, significant main effects were observed for time (F = 11.33, *p* < 0.05, ηp^2^ = 0.24), group (F = 8.49, *p* < 0.05, ηp^2^ = 0.19), and gender (F = 12.95, *p* < 0.05, ηp^2^ = 0.27). No significant interactions among time, gender, and group were found (*p* > 0.05). For the AET, significant main effects were found for time (F = 31.15, *p* < 0.05, ηp^2^ = 0.46) and group (F = 7.84, *p* < 0.05, ηp^2^ = 0.18). A significant group-by-gender interaction (F = 5.17, *p* < 0.05, ηp^2^ = 0.13) was also observed (Table 4).

Table 4 presents the changes in the DLLT, AMST and AET across the baseline, post-training, and follow-up assessments for both the ADIM and ADIM+BFR groups. The ADIM group demonstrated no significant changes in any of the measured variables (DLLT, AMST, and AET) across the three time points (*p* > 0.05). Conversely, the ADIM+BFR group exhibited significant time-dependent differences in both the DLLT (females: F = 9.54, *p* < 0.05, ηp^2^ = 0.51; males: F = 45.82, *p* < 0.05, ηp^2^ = 0.84) and AET (females: F = 12.29, *p* < 0.05, ηp^2^ = 0.58; males: F = 7.15, *p* < 0.05, ηp^2^ = 0.44). Specifically, post hoc analysis revealed significant differences in the DLLT between baseline and both the post-training and follow-up assessments for both female and male participants in the ADIM+BFR group (*p* < 0.05). For the AET, significant differences compared to baseline were observed only at the follow-up assessment for both female and male participants (*p* < 0.05). Furthermore, significant differences between the ADIM+BFR and ADIM groups were observed in females at both the post-training (DLLT: *t* = −2.81, *p* < 0.05, Cohen’s *d* = 1.26; AMST: *t* = 2.33, *p* < 0.05, Cohen’s *d* = 1.04; AET: *t* = 3.36, *p* < 0.05, Cohen’s *d* = 1.50) and follow-up (DLLT: *t* = −3.28, *p* < 0.05, Cohen’s *d* = 1.47; AMST: *t* = 2.33, *p* < 0.05, Cohen’s *d* = 1.04; AET: *t* = 3.92, *p* < 0.05, Cohen’s *d* = 1.75) assessments. In males, the ADIM+BFR group showed significant differences compared to the ADIM group in the DLLT post-training (*t* = −3.67, *p* < 0.05, Cohen’s *d* = 1.42) and at follow-up (*t* = −2.73, *p* < 0.05, Cohen’s *d* = 1.22), and in the AMST at follow-up (*t* = 2.43, *p* < 0.05, Cohen’s *d* = 1.09).

## 4. Discussion

To our knowledge, few if any prior studies have applied BFR to core stability training, as most BFR research has focused on limb muscles. This study addresses that gap by investigating the effects of ADIM combined with BFR training on measures of deep core muscle function, explicitly focusing on TrA thickness, contraction ability, and the performance of core muscles in sedentary individuals. The findings revealed statistically significant enhancements across multiple parameters within the ADIM+BFR intervention group, while the ADIM-only group exhibited no substantive changes. These results suggest that ADIM+BFR may effectively improve deep abdominal muscle function in sedentary populations. While some improvements in abdominal muscle characteristics and performance were observed during the follow-up period, it is important to note that not all gains were maintained. This suggests that BFR+ADIM training can induce positive improvements in TrA, but continued exercise or lifestyle modifications are likely necessary to sustain these benefits long-term.

The maintenance of TrA thickness holds significant clinical relevance given its pivotal role in core stability and postural control. Consequently, sustained hypertrophy in this muscle may translate to long-term enhancements in functional capacity and a reduction in musculoskeletal injury risk [25]. The ADIM is renowned for maximizing TrA activation while maintaining a neutral spinal posture [26]. Fundamentally, the ADIM improves the neuromuscular control of deep trunk muscles, particularly the TrA, which plays a crucial role in lumbar segmental stability [27,28,29]. However, the technical complexity of the ADIM may hinder accurate TrA activation, and improper execution can lead to the compensatory activation and hypertrophy of the EO muscle [12]. The findings of this study revealed a significant post-training augmentation of TrA contracted thickness in the ADIM+BFR group, a result consistent with the established literature demonstrating the efficacy of BFR training in inducing muscle hypertrophy [30,31].

Furthermore, this study found that the beneficial effects persisted after training cessation, resulting in sustained enhancements in TrA contraction capacity and neuromuscular activation efficiency. The absence of significant changes in the ADIM-only group further supports the crucial role of BFR in facilitating muscle contraction improvements. This finding corroborates a previous study demonstrating the positive influence of BFR on muscle adaptation and neuromuscular activation [32]. Yasuda et al. demonstrated that adding BFR to low-intensity resistance exercise led to strength gains comparable to high-intensity training, which aligns with our finding that low-load ADIM exercise combined with BFR can produce significant improvements. The conditions are analogous to using BFR to boost a low-intensity stimulus. However, Yasuda’s protocol involved dynamic resistance exercises for limbs, whereas the current study involved an isometric core exercise. Nevertheless, it is evident that the ADIM+BFR training program resulted in modifications and improvements in the neuromuscular control of deep core muscles. However, the maintenance of these benefits likely requires ongoing physical activity. For sedentary populations, who are at an increased risk of muscle dysfunction and neuromuscular decline, sustained exercise programs are crucial for preserving functional gains achieved through interventions like ADIM+BFR.

A significant reduction in the disparity between resting and contracted EO thickness was observed post-ADIM+BFR training, with this effect remaining demonstrable one week after the training period. Specific studies have demonstrated a correlation between core muscle imbalances, characterized by overactive EO muscles and underactive deep core muscles (diaphragm, TrA, and IO), and the development of lumbar spine instability, which can lead to increased fatigue, reduced endurance, and a heightened risk of low back pain [33,34,35,36]. Consistent with the intended function of the ADIM, which selectively activates deep core muscles, particularly the TrA, while minimizing EO activation [21,37], ADIM+BFR training can achieve the same effect of not causing excessive EO activation. This study provides evidence that eight sessions of ADIM+BFR training can significantly and rapidly activate the TrA while preventing compensatory EO activation in sedentary populations. Our findings highlight BFR’s potential to enhance low-intensity core training in young sedentary adults. It is speculated that the combination of BFR with the ADIM may offer a promising therapeutic intervention for individuals experiencing difficulties in performing the ADIM, such as those with low back pain or the elderly.

Abdominal muscle performance assessments in sedentary populations reveal statistically significant enhancements in the DLLT, AMST and AET within the ADIM+BFR intervention group, indicating that BFR-combined training effectively augments abdominal muscle performance, encompassing stability, strength, and endurance in sedentary populations. This result aligns with the principles of core muscle training, which posits that increasing core stability through strengthening local muscles (stabilizer), particularly the TrA, plays a key role in overall abdominal muscle performance [38,39]. The ADIM-only group exhibited no significant alterations in muscle thickness parameters, nor did they show significant improvements in abdominal muscle function. These results suggest that ADIM alone, without BFR, might not provide sufficient stimulus for functional adaptations in sedentary individuals within the study’s timeframe. Potential explanations for this include insufficient training intensity, limited training duration, or inherent limitations of the ADIM for inducing muscle hypertrophy in this population. Therefore, further research is needed to validate these applications and to examine if a more extended training period or higher training intensity would be necessary to observe significant changes with the ADIM alone.

Potential gender-specific differences in muscle morphology, function, metabolism, fatigability, and contractile properties have been reported across numerous studies; however, these differences arise from complex factors, including neural, muscular, motor learning, genetic, and hormonal influences. Thus, these findings remain controversial [40,41,42,43,44,45,46]. Prior studies have indicated gender-based differences in quadriceps femoris fatigability during combined isotonic knee extension exercises. Conversely, the ADIM-only group displayed no significant alterations in muscle thickness parameters. These findings suggest potential benefits for individuals experiencing low back pain or elderly individuals with BFR [47]. This study observed some gender-specific differences in muscle thickness, muscle contraction ability, and activation following the ADIM+BFR intervention. Male participants showed significant enhancements in both TrA resting and contracted thickness and IO contracted thickness, while female participants primarily exhibited changes in TrA contracted thickness. Female participants demonstrated significant improvements across all abdominal muscle performance tests, whereas males mainly showed enhancements in the DLLT and AMST. These differences may be attributed to inherent gender-related physiological variations in muscle responsiveness to BFR training, with males potentially exhibiting a higher response in contraction thickness and females in muscle performance, possibly reflecting inherent gender-based differences in the demands of abdominal muscle performance in sedentary populations. These gender differences may be partly explained by known sex-specific characteristics in muscle physiology, such as hormonal influences on muscle growth and activation patterns. More specifically, testosterone levels in males could contribute to greater hypertrophic responsiveness, whereas estrogen in females might influence muscle stiffness or endurance differently. While our results suggested some sex-specific responses, these differences were not the central focus of the study, and our sample size was not powered for definitive conclusions on gender interactions.

### Study Limitations

Several limitations must be considered when interpreting the results of this study. Firstly, using inflatable cuffs for BFR introduces potential risks and discomfort. While the study effectively demonstrated the benefits of BFR combined with ADIM exercise on TrA thickness and activation, it is essential to acknowledge the factors that can influence discomfort, such as applied pressure, cuff dimensions, cuff composition, participant gender, and training to volitional fatigue [48]. Additionally, BFR carries potential risks, including venous thrombus, pulmonary embolism, and rhabdomyolysis [49]. Although no adverse events occurred in this study, future research should investigate methods to mitigate cuff-induced discomfort and improve overall safety. This should include a thorough examination of individual patient risk factors and the optimization of application techniques. Secondly, it is acknowledged that the 4-week intervention implemented was relatively short, and although adequate for observing neuromuscular activation improvements and early strengthening and endurance gains, it was probably insufficient for measurable hypertrophic changes in muscle size. Future studies should incorporate longer training periods and follow-up assessments to evaluate the durability of improvements and whether additional training leads to significant muscle atrophy.

Moreover, the limited sample size and the convenience of recruiting only university students as the first stage of the planned research project may restrict the generalizability and applicability of our findings. Future studies should aim to expand the sample size and include diverse populations, such as individuals with low back pain, for validation. Additionally, this study did not delve into the specific mechanisms underlying BFR training. Future research could incorporate electrophysiological and neuromuscular approaches to explore the neural control mechanisms through which BFR training affects deep core muscles.

## 5. Conclusions

In conclusion, this study demonstrates that the ADIM combined with BFR training can significantly improve sedentary individuals’ TrA thickness, contraction ability, and overall abdominal muscle performance. These findings suggest that ADIM+BFR represents a potentially valuable intervention for enhancing core muscle function in this population, particularly for increasing TrA activation and strength. Furthermore, it is observed that such improvement was evident for both male and female participants. However, baseline differences in muscle thickness were evident between genders, which may have subsequently impacted the magnitude of the response.

Despite the relatively positive outcome, the limitations of this study, such as the sample size and specific population due to convenient recruitment, should be considered when interpreting the results, and its applicability to the general population will require further studies to determine. The results of this study offer an alternative approach for developing effective interventions aimed at enhancing deep core muscle function in sedentary individuals.

## Figures and Tables

**Table 1:** Characteristics of participants in ADIM+BFR and ADIM groups.

	ADIM + BFR		ADIM	
	Female	Male	Female	Male
Number	10	10	10	10
Age (years)	22.5 ± 3.5	22.2 ± 2.2	22.8 ± 3.4	23.8 ± 3.1
Weight (kg)	54.4 ± 13.9	71.5 ± 9.1	52.9 ± 7.5	72.9 ± 9.5
Height (cm)	165.8 ± 4.2	175.2 ± 5.3	163.8 ± 2.2	177.2 ± 7.3
BMI	22.4 ± 4.1	24.1 ± 2.2	21.4 ± 3.5	23.42
Sedentary behavior (minutes/day)	885.64 ± 239.84	871.02 ± 266.52	866.35 ± 271.13	874.92 ± 285.21
Duration of regular exercise (minutes/week)	26.00 ± 23.16	34.00 ± 16.27	29.50 ± 18.19	30.00 ± 19.80

**Table 2:** Serial changes in the thickness of external oblique (EO), internal oblique (IO), and transversus abdominis (TrA) muscles in ADIM+BFR and ADIM groups from baseline to post-training and follow-up.

								Within-Subjects Comparison				Between-Subjects Comparison		
					Data Collection Points (Time)			Time	Time × Group	Time × Gender	Time × Group × Gender	Group	Gender	Group × Gender
Muscle	Action	Group	Gender	N	Baseline	Post-Training	Follow-Up	F Value	F Value	F Value	F Value	F Value	F Value	F Value
EO	ADIM	ADIM+BFR	F	10	4.81 ± 1.23	4.12 ± 0.47	4.57 ± 0.63	0.79	2.82	3.35	1.11	1.58	43.87 *	4.59 *
			M	10	7.10 ± 1.54	7.34 ± 1.27	7.10 ± 1.39							
		ADIM	F	10	4.74 ± 0.82	4.92 ± 1.45	4.64 ± 1.20							
			M	10	6.39 ± 1.41	6.51 ± 1.21	6.52 ± 1.14							
	Resting	ADIM+BFR	F	10	5.35 ± 1.51	5.26 ± 0.86	5.38 ± 0.93	4.02 *	0.49	1.68	0.02	3.07	35.19 *	2.27
			M	10	7.19 ± 1.09	8.29 ± 1.48	8.43 ± 1.67							
		ADIM	F	10	5.03 ± 0.76	5.31 ± 1.17	5.37 ± 1.13							
			M	10	6.37 ± 1.63	7.10 ± 1.29	7.31 ± 1.34							
IO	ADIM	ADIM+BFR	F	10	4.75 ± 1.56	5.52 ± 1.14	5.45 ± 0.90	12.13 *	1.73	1.98	1.15	1.26	19.04 *	1.65
			M	10	7.32 ± 1.82	9.35 ± 1.80 ^‡^	7.67 ± 1.01							
		ADIM	F	10	4.77 ± 1.21	5.55 ± 1.68	5.65 ± 1.00							
			M	10	6.11 ± 1.80	7.29 ± 1.54	7.27 ± 1.54							
	Resting	ADIM+BFR	F	10	4.70 ± 1.76	4.60 ± 1.07	4.70 ± 1.08	3.45 *	1.45	2.63	1.07	0.75	17.89 *	1.41
			M	10	7.25 ± 1.09	8.34 ± 1.66	6.93 ± 1.41							
		ADIM	F	10	4.64 ± 1.35	4.95 ± 1.51	4.91 ± 1.38							
			M	10	6.68 ± 1.93	7.90 ± 1.69	6.70 ± 1.36							
TrA	ADIM	ADIM+BFR	F	10	3.37 ± 0.78	4.46 ± 1.16 ^†,‡^	4.17 ± 0.98	6.96 *	14.34 *	0.48	5.82 *	14.17 *	0.23	0.05
			M	10	3.07 ± 1.08	4.90 ± 1.50 ^†,‡^	4.57 ± 0.71 ^†,‡^							
		ADIM	F	10	2.80 ± 0.36	3.12 ± 1.05	3.33 ± 1.26							
			M	10	2.73 ± 1.25	2.82 ± 0.95	2.91 ± 0.70							
	Resting	ADIM+BFR	F	10	2.76 ± 0.78	3.19 ± 1.16	2.70 ± 1.11	2.21	5.28 *	2.66	3.39 *	2.30	0.12	0.04
			M	10	2.59 ± 0.81	3.10 ± 0.88 ^†^	2.60 ± 0.82							
		ADIM	F	10	2.39 ± 0.46	2.51 ± 0.74	2.62 ± 0.86							
			M	10	3.06 ± 1.05	2.24 ± 0.52	2.10 ± 0.55							

* Significant difference (*p* < 0.05), as determined by a mixed-model two-way analysis of variance. ^‡^ Significant difference (*p* < 0.05) compared to baseline values, as determined by Bonferroni post hoc tests. ^†^ Significant difference (*p* < 0.05) between the ADIM+BFR and ADIM groups in both females (F) and males (M), as determined by independent *t*-tests.

**Table 3:** Time-course changes in the contraction and activation ratio of external oblique (EO), internal oblique (IO), and transversus abdominis (TrA) muscles in ADIM+BFR and ADIM groups from baseline to post-training and follow-up.

								Within-Subjects Comparison				Between-Subjects Comparison		
					Data Collection Time Points			Time	Time × Group	Time × Gender	Time × Gender × Group	Group	Gender	Group × Gender
Index	Muscle	Group	Gender	N	Baseline	Post-Training	Follow-Up	F Value	F Value	F Value	F Value	F Value	F Value	F Value
CR	EO	ADIM+BFR	F	10	0.91 ± 0.11	0.79 ± 0.11 ^‡^	0.86 ± 0.09	2.28	2.76	2.76	2.40	1.03	0.12	1.27
			M	10	0.93 ± 0.10	0.90 ± 0.15	0.85 ± 0.10 ^‡^							
		ADIM	F	10	0.95 ± 0.09	0.92 ± 0.15	0.86 ± 0.11							
			M	10	0.85 ± 0.10	0.92 ± 0.06	0.90 ± 0.08							
	IO	ADIM+BFR	F	10	1.04 ± 0.18	1.21 ± 0.14 ^‡^	1.20 ± 0.27 ^‡^	4.46 *	1.97	0.82	0.34	1.00	0.45	0.09
			M	10	1.03 ± 0.10	1.15 ± 0.23	1.17 ± 0.25							
		ADIM	F	10	1.04 ± 0.10	1.13 ± 0.11	1.14 ± 0.16							
			M	10	1.11 ± 0.19	1.07 ± 0.10	1.09 ± 0.11							
	TrA	ADIM+BFR	F	10	1.24 ± 0.16	1.48 ± 0.42	1.68 ± 0.53 ^†,‡^	13.54 *	6.98 *	0.77	0.45	12.77 *	1.35	0.04
			M	10	1.20 ± 0.22	1.61 ± 0.31 ^†,‡^	1.89 ± 0.53 ^†,‡^							
		ADIM	F	10	1.20 ± 0.20	1.24 ± 0.13	1.26 ± 0.13							
			M	10	1.27 ± 0.40	1.24 ± 0.23	1.39 ± 0.15							
Diff	EO	ADIM+BFR	F	10	0.54 ± 0.65	1.14 ± 0.81 ^†,‡^	0.81 ± 0.53	2.85	2.75	1.55	2.40	2.19	1.56	0.17
			M	10	0.69 ± 0.76	0.95 ± 0.28	1.33 ± 0.04 ^‡^							
		ADIM	F	10	0.28 ± 0.52	0.39 ± 0.64	0.73 ± 0.59							
			M	10	0.98 ± 0.70	0.58 ± 0.45	0.80 ± 0.71							
	IO	ADIM+BFR	F	10	−0.05 ± 0.72	−0.92 ± 0.59 ^‡^	−0.75 ± 0.98 ^‡^	4.91 *	1.46	0.23	0.29	0.37	0.00	0.03
			M	10	−0.07 ± 0.66	−1.01 ± 0.20	−0.75 ± 0.33							
		ADIM	F	10	−0.13 ± 0.49	−0.60 ± 0.50	−0.73 ± 0.83							
			M	10	−0.43 ± 0.83	−0.39 ± 0.63	−0.56 ± 0.78							
	TrA	ADIM+BFR	F	10	−0.61 ± 0.41	−1.27 ± 0.99	−1.47 ± 0.92 ^†,‡^	14.32 *	8.02 *	0.44	1.77	17.22 *	1.72	0.40
			M	10	−0.48 ± 0.66	−1.80 ± 0.89 ^†,‡^	−1.97 ± 0.69 ^†,‡^							
		ADIM	F	10	−0.41 ± 0.40	−0.61 ± 0.43	−0.71 ± 0.52							
			M	10	−0.66 ± 0.60	−0.58 ± 0.59	−0.81 ± 0.31							
PAR	EO	ADIM+BFR	F	10	−0.10 ± 0.09	−0.27 ± 0.10 ^†,‡^	−0.23 ± 0.10 ^‡^	11.36 *	5.84 *	2.52	1.38	3.87	0.87	0.92
			M	10	−0.07 ± 0.07	−0.17 ± 0.19 ^‡^	−0.22 ± 0.12 ^‡^							
		ADIM	F	10	−0.08 ± 0.07	−0.14 ± 0.10	−0.17 ± 0.09							
			M	10	−0.15 ± 0.12	−0.10 ± 0.07	−0.14 ± 0.08							
	IO	ADIM+BFR	F	10	0.01 ± 0.15	0.04 ± 0.14	0.04 ± 0.15	0.98	1.22	2.40	1.08	0.12	0.10	0.28
			M	10	0.03 ± 0.08	0.05 ± 0.14	0.05 ± 0.14							
		ADIM	F	10	0.02 ± 0.07	0.07 ± 0.10	0.09 ± 0.12							
			M	10	0.10 ± 0.16	0.04 ± 0.07	0.05 ± 0.08							
	TrA	ADIM+BFR	F	10	0.21 ± 0.19	0.37 ± 0.28	0.44 ± 0.30 ^†^	10.32 *	5.39 *	1.63	0.75	14.81 *	4.70 *	0.08
			M	10	0.20 ± 0.16	0.46 ± 0.19 ^†,‡^	0.69 ± 0.28 ^†,‡^							
		ADIM	F	10	0.17 ± 0.18	0.17 ± 0.12	0.21 ± 0.09							

Contraction ratio (CR), the difference in muscle thickness between resting and ADIM contraction (Diff), and preferential activation ratio (PAR) were calculated as indices of muscle activity. *: *p* < 0.05, tested by a mixed model two-way analysis of variance; ^‡^: tested by Bonferroni compared with baseline value; ^†^: *p* < 0.05, there is significant difference between ADIM+BFR and ADIM groups in both females (F) and males (M), as determined by independent *t*-tests.

**Table 4:** Time-course changes in abdominal muscle functional test in ADIM+BFR and ADIM groups from baseline to post-training and follow-up.

							Within-Subjects Comparison				Between-Subjects Comparison		
				Data Collection Time Points			Time	Time × Group	Time × Gender	Time × Gender × Group	Group	Gender	Group × Gender
Test	Group	Gender	N	Baseline	Post-Training	Follow-Up	F Value	F Value	F Value	F Value	F Value	F Value	F Value
DLLT	ADIM+BFR	F	10	73.25 ± 7.36	59.50 ± 12.24 ^†,‡^	57.50 ± 13.23 ^†,‡^	36.05 *	12.71 *	1.84	0.05	15.52 *	1.69	0.68
(degree)		M	10	74.25 ± 3.74	59.00 ± 7.75 ^†,‡^	54.00 ± 12.14 ^†,‡^							
	ADIM	F	10	74.75 ± 4.63	72.50 ± 7.99	73.25 ± 7.46							
		M	10	73.25 ± 7.64	68.25 ± 5.01	65.50 ± 5.50							
AMST	ADIM+BFR	F	10	22.81 ± 6.46	24.98 ± 4.61 ^†^	24.65 ± 4.86 ^†^	11.33 *	0.55	0.39	0.35	8.49 *	12.95 *	0.22
(lb)		M	10	25.35 ± 4.70	29.04 ± 2.88	28.61 ± 3.14 ^†^							
	ADIM	F	10	19.15 ± 2.88	21.08 ± 2.57	20.77 ± 2.04							
		M	10	23.73 ± 4.81	26.12 ± 4.45	24.90 ± 3.68							
AET	ADIM+BFR	F	10	72.50 ± 55.08	89.30 ± 34.05 ^†^	114.00 ± 41.39 ^†,‡^	31.15 *	2.88	1.16	1.62	7.84 *	0.19	5.17 *
(second)		M	10	60.30 ± 21.18	80.00 ± 11.59	92.00 ± 22.79 ^‡^							
	ADIM	F	10	46.30 ± 12.07	50.90 ± 11.99	58.30 ± 17.40							
		M	10	56.30 ± 19.59	79.30 ± 29.96	84.20 ± 26.08							

Double leg lowering test (DLLT), abdominal muscle strength test (AMST) and abdominal endurance test (AET) were measured as core muscle function. *: *p* < 0.05, tested by a mixed model two-way analysis of variance; ^‡^ Significant difference (*p* < 0.05) compared to baseline values, as determined by Bonferroni post hoc tests. ^†^ Significant difference (*p* < 0.05) between the ADIM+BFR and ADIM groups in both female (F) and male (M), as determined by independent *t*-tests.

## Data Availability

The original contributions presented in this study are included in the article. Further inquiries can be directed to the corresponding author(s).

## Abbreviations

The following abbreviations are used in this manuscript:

ADIM	abdominal drawing-in maneuver
AET	abdominal endurance test
AMST	abdominal muscle strength test
BFR	blood flow restriction
CR	contraction ratio
Diff	difference in contraction thickness
DLLT	double leg lowering test
EO	external oblique
ICC	intraclass correlation coefficient
IO	internal oblique
PAR	preferential activation ratio
TrA	transversus abdominis
